# First person – Gideon Hughes

**DOI:** 10.1242/dmm.047415

**Published:** 2020-10-16

## Abstract

First Person is a series of interviews with the first authors of a selection of papers published in Disease Models & Mechanisms, helping early-career researchers promote themselves alongside their papers. Gideon Hughes is first author on ‘Machine learning discriminates a movement disorder in a zebrafish model of Parkinson's disease’, published in DMM. Gideon conducted the research described in this article while a PhD student in Betsy Pownall's lab at the University of York, York, UK. He is now a postdoc in the lab of Henry Roehl at the University of Sheffield, Sheffield, UK, using the zebrafish as a model organism to study human disease and tissue regeneration, combining his research with his interest in computer science.


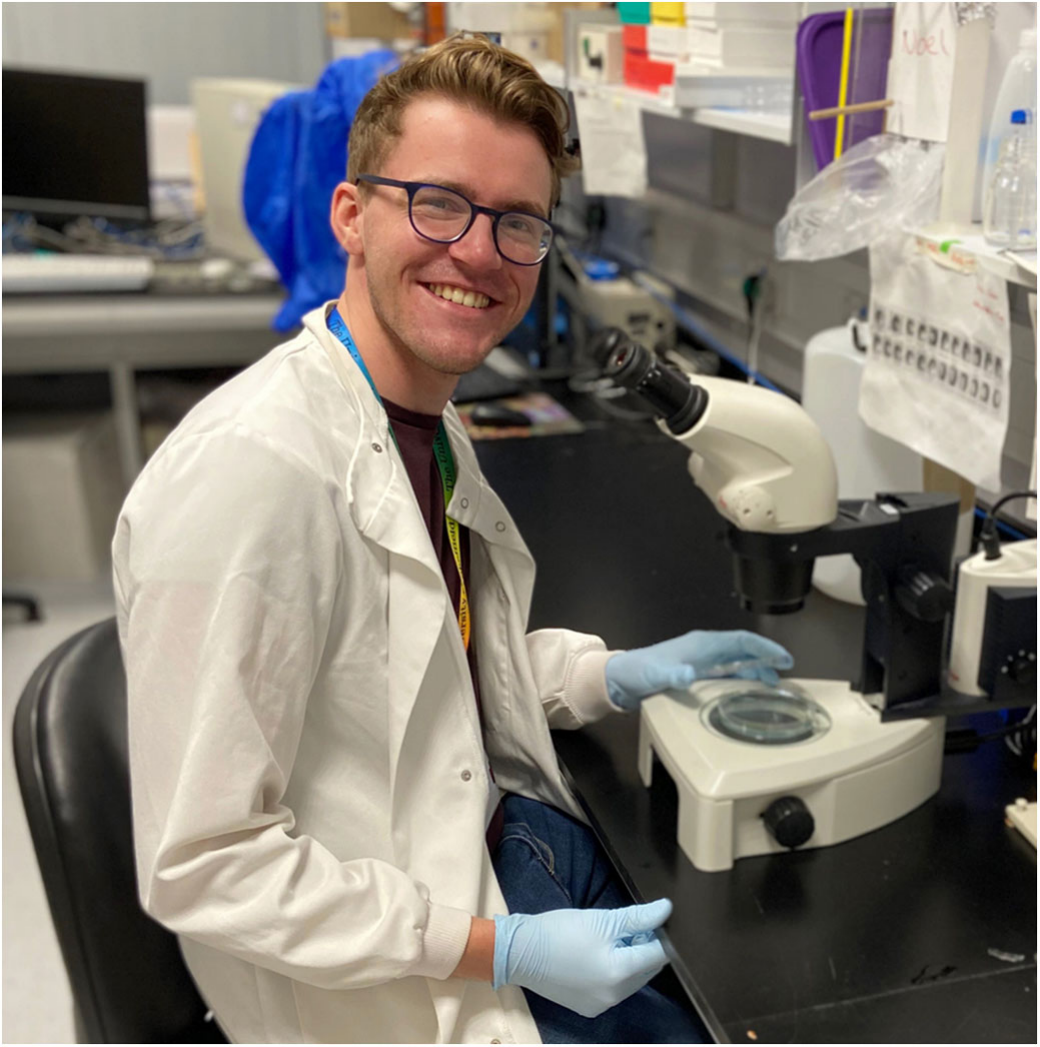


**Gideon Hughes**

**How would you explain the main findings of your paper to non-scientific family and friends?**

Parkinson's disease (PD) is caused by a progressive loss of dopaminergic neurons in a region of the brain called the substantia nigra. A loss of motor control occurs in patients and there is currently no cure for PD. To identify potential treatments, drug screening using a suitable animal model is a very valuable step before reaching clinical trials. Zebrafish are a vertebrate model organism well suited for drug screening, and gene editing can be used in zebrafish to create mutations in genes that cause PD in humans. To model PD in zebrafish, we used gene editing to mutate *dj-1* (a gene known to cause PD in people) and found a reduction in the number of dopaminergic neurons in the fish brain, a feature consistent with the human condition. Usually, researchers use zebrafish larvae for these kinds of studies, but since PD is a disease associated with old age, it made more sense for us to develop an adult model. We wanted to know if our fish model of PD showed a movement disorder similar to those seen in patients with PD. To do this, we developed a protocol that uses machine learning to recognise movement disorder in adult *dj-1* mutants. There is future potential to test new PD therapies on adult *dj-1* mutants and to see whether the movement disorder is rescued, using the evolved algorithm.

**What are the potential implications of these results for your field of research?**

In the field of PD research, our *dj-1* mutant line may be used by other researchers as part of a therapeutic pipeline for drug discovery. Our data, from a transcriptomic analysis, point to changes in cell bioenergetics that may help direct future research in how this contributes to PD pathology. There is also the potential of other researchers adopting our method of capturing adult swimming movements, including distinct tail bend angles, to calculate differences in the features of movement. This would not require the computer science used to evolve classifiers, so could be more widely applied as it just needs the freely available ShadowFish tracking software. The machine learning could be adopted to recognise more subtle movement disorders in other zebrafish models of neuro or muscular degenerative diseases.

**What are the main advantages and drawbacks of the model system you have used as it relates to the disease you are investigating?**

Many labs use zebrafish to model human disease as they share many of the same genes with humans, and mutants are easily generated. A drawback of using zebrafish as a model for PD is that these studies are usually done using embryos and/or larvae. The main advantage of our study is that we used adult fish and we developed a method to identify a recognised symptom of PD, a movement disorder, by using machine learning to distinguish fish mutant for a PD gene (*dj-1*) from wild-type fish. The drawback is that using adults is not high throughput, and this would mean that any chemical screen or drug development using our analysis would need to be focused on a particular set of candidate drugs. Still, this could make a valuable contribution to pre-clinical testing.

“The contribution of machine learning to the analysis of these fish was remarkable, and I wouldn't be surprised if machine learning could be applied to a range of biological problems to provide some objective solutions.”

**What has surprised you the most while conducting your research?**

I was surprised by the efficiency of using genome editing in the zebrafish to create models of human disease. The advent of CRISPR/Cas9 technology in model organisms has opened the door to modelling any disease with a known genetic basis. Being able to recognise features of the human disease in a different organism, like a fish, is also surprising. The contribution of machine learning to the analysis of these fish was remarkable, and I wouldn't be surprised if machine learning could be applied to a range of biological problems to provide some objective solutions.

**Figure:**
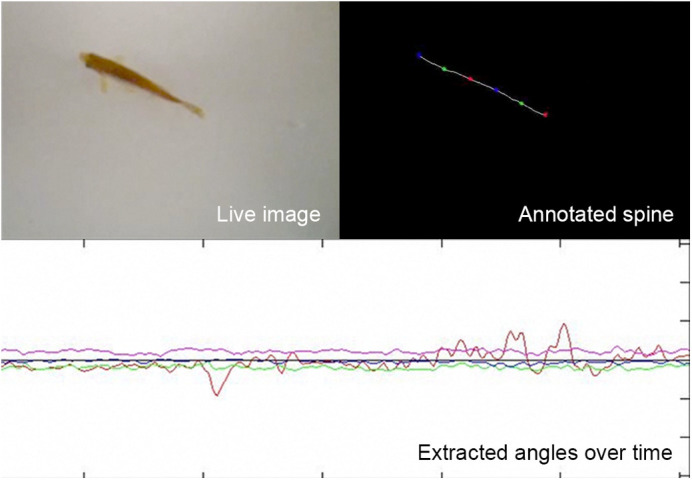
**Using our ShadowFish tracking software to process a recording, the spine of the zebrafish is annotated and the angles along the spine are extracted for each frame.**

**Describe what you think is the most significant challenge impacting your research at this time and how will this be addressed over the next 10 years?**

A significant challenge is identifying the exact molecular mechanism that is disrupted in individual people with PD. Inherited cases of PD, caused by mutations in different genes, can cause the disruption of distinct molecular processes that underlie each person's disease. This could mean that different patients have dissimilar responses to the same treatment. We used RNA-seq here to identify changes in gene expression in the *dj-1* mutant zebrafish brain to further investigate disrupted molecular mechanisms. In the future, transcriptomic analyses could be carried out on dopaminergic neurons, generated from human stem cells, harbouring mutations in PD-associated genes. The results of these analyses would help address the issue of molecular pathology in individual cases.

**What changes do you think could improve the professional lives of early-career scientists?**

Collaborations with other researchers, from a range of fields, could help improve the professional lives of early-career scientists. During my PhD I got to collaborate with both the Currie lab at Monash University and Dr Michael Lones from Heriot-Watt University. Visiting the Currie lab in Australia was a great opportunity for me, as it allowed me to work in an excellent zebrafish research facility and learn from a group of highly experienced zebrafish researchers with a longstanding focus on using fish to study human disease. Our collaboration with Michael Lones in Edinburgh provided us with top-level computer science involvement that made it possible to evolve classifiers that recognise PD fish using the raw movement data, thereby removing any bias and increasing the accuracy of our approach. Both collaborations have contributed to the impact of our research.

**What's next for you?**

I have recently started a postdoctoral research position at the University of Sheffield. My research is now focused on using zebrafish as a model organism to study tissue regeneration. I hope to combine my interest in computer science with the study of regeneration and I am currently working to create a light-sheet microscope that can automatically move the sample to keep any migrating cells in the field of view.

**What did you gain from your PhD?**

The training I got from this highly interdisciplinary PhD project has allowed me to develop my ability to think critically about scientific problems in both molecular genetics and computational analyses, while allowing me to contribute to the understanding of a devastating human disease. Having two supervisors from such different areas kept our lab meetings both interesting and challenging!
